# Predicting streptococcal pharyngitis in adults in primary care: a systematic review of the diagnostic accuracy of symptoms and signs and validation of the Centor score

**DOI:** 10.1186/1741-7015-9-67

**Published:** 2011-06-01

**Authors:** Jolien Aalbers, Kirsty K O'Brien, Wai-Sun Chan, Gavin A Falk, Conor Teljeur, Borislav D Dimitrov, Tom Fahey

**Affiliations:** 1 HRB Centre for Primary Care Research, Department of General Practice, RCSI Medical School, Dublin, Republic of Ireland; 2 Radboud University Nijmegen Medical Centre, Nijmegen, The Netherlands; 3 Department of Public Health and Primary Care, Trinity Centre, AMNCH, Dublin, Republic of Ireland

## Abstract

**Background:**

Stratifying patients with a sore throat into the probability of having an underlying bacterial or viral cause may be helpful in targeting antibiotic treatment. We sought to assess the diagnostic accuracy of signs and symptoms and validate a clinical prediction rule (CPR), the Centor score, for predicting group A β-haemolytic streptococcal (GABHS) pharyngitis in adults (> 14 years of age) presenting with sore throat symptoms.

**Methods:**

A systematic literature search was performed up to July 2010. Studies that assessed the diagnostic accuracy of signs and symptoms and/or validated the Centor score were included. For the analysis of the diagnostic accuracy of signs and symptoms and the Centor score, studies were combined using a bivariate random effects model, while for the calibration analysis of the Centor score, a random effects model was used.

**Results:**

A total of 21 studies incorporating 4,839 patients were included in the meta-analysis on diagnostic accuracy of signs and symptoms. The results were heterogeneous and suggest that individual signs and symptoms generate only small shifts in post-test probability (range positive likelihood ratio (+LR) 1.45-2.33, -LR 0.54-0.72). As a decision rule for considering antibiotic prescribing (score ≥ 3), the Centor score has reasonable specificity (0.82, 95% CI 0.72 to 0.88) and a post-test probability of 12% to 40% based on a prior prevalence of 5% to 20%. Pooled calibration shows no significant difference between the numbers of patients predicted and observed to have GABHS pharyngitis across strata of Centor score (0-1 risk ratio (RR) 0.72, 95% CI 0.49 to 1.06; 2-3 RR 0.93, 95% CI 0.73 to 1.17; 4 RR 1.14, 95% CI 0.95 to 1.37).

**Conclusions:**

Individual signs and symptoms are not powerful enough to discriminate GABHS pharyngitis from other types of sore throat. The Centor score is a well calibrated CPR for estimating the probability of GABHS pharyngitis. The Centor score can enhance appropriate prescribing of antibiotics, but should be used with caution in low prevalence settings of GABHS pharyngitis such as primary care.

## Background

Upper respiratory tract infections such as acute pharyngitis represent a substantial portion of the cases seen in primary care [1]. Although the cause of acute pharyngitis in the majority of patients is viral, approximately 5% to 17% is caused by a bacterial infection, often β-haemolytic streptococci [2]. A number of serotypes of β-haemolytic streptococci can cause pharyngitis in humans, however, antibiotics are only recommended in US and UK guidelines for treating patients with group A β-haemolytic streptococcal (GABHS) pharyngitis [3,4]. Antibiotics reduce the risk of complications (for example, peritonsillar abscess, bacteraemia, acute glomerulonephritis and rheumatic fever), as well as reducing the duration of symptoms and spread of the disease [5-7].

Throat cultures are currently considered to be the 'reference standard' for the diagnosis of streptococcal pharyngitis [8,9]. This test has a number of limitations in practice; it is relatively expensive; the laboratory tests take 1-2 days leading to delays in starting treatment; and excessive false positive results in asymptomatic pharyngeal carriers may lead to over treatment [10,11]. To enhance the appropriate prescribing of antibiotics without performing cultures on all patients a number of clinical prediction rules (CPRs) have been developed over the last 40 years to distinguish streptococcal pharyngitis from pharyngitis by other causes [12-15]. CPRs are evidence-based tools that allow clinicians to stratify patients according to their probability of having a particular disorder. They can also be used to provide a rational basis for treatment.

The most widely recognised CPR for GABHS pharyngitis is the Centor score [16]. The Centor score consists of four signs and symptoms (Table 1) and is recommended in clinical guidelines from the American College of Physicians-American Society of Internal Medicine (ACP/ASIM) and Centers for Disease Control and Prevention (CDC) in the US. The ACP/ASIM recommends (a) empirical antibiotic treatment of adults with at least three of four Centor criteria and no treatment for all others; or (b) empirical treatment of adults with all four criteria, rapid antigen detection test (RADT) of patients with three or two criteria, and subsequent treatment of those with positive test results and no treatment for all others [17]. In the UK, the National Institute for Health and Clinical Excellence (NICE) recommend that clinicians consider immediate treatment with antibiotics for patients who have three or more Centor criteria [4]. A modified version of the Centor criteria is also used in New Zealand as part of a guideline for sore throat management [14,18].

**Table 1:** The Centor score

Symptoms	Points	Score	Post-test probability
Tonsillar exudates	1	0	2.5%

Tender anterior cervical adenopathy	1	1	6.5%

Absence of cough	1	2	15.4%

History of fever (> 38.0°C)	1	3	31.6%

		4	55.7%

Patients receive a point for the presence or absence of signs and symptoms. Each patient is assigned a score between 0 and 4 which is associated with a post-test probability as calculated by Centor and colleagues [16]. (The post-test probability values presented are the mean of the original probability intervals reported by Centor and colleagues.)

The pretest probability of GABHS pharyngitis is reported to peak between the ages of 5 and 10 years [15]. The prevalence in children is reported to be around 20% to 25% while in adults it is between 5% to 10% [12]. This review will focus on adults (≥ 15 years of age), the age group of the cohort in which the Centor score was derived.

Although a considerable amount of research has already been devoted to streptococcal pharyngitis, it remains unclear which symptoms and signs have the most discriminatory power and whether the most widely recognised rule, the Centor score, is valid in a range of clinical settings. The aim of this systematic review was to analyse the current evidence on the usefulness of individual signs and symptoms in assessing the risk of streptococcal pharyngitis in adults, to assess the diagnostic accuracy of the Centor score as a decision rule for antibiotic treatment (discrimination analysis) and to perform a meta-analysis on validation studies of the Centor score (calibration analysis).

## Methods

### Data sources and searches

An electronic search was performed using a search filter developed by Haynes *et al. *[19,20]. This preset filter (search string: (predict*[tiab] OR predictive value of tests[mh] OR scor*[tiab] OR observ*[tiab] OR observer variation[mh]) is available in the PubMed database and has a reported sensitivity of 96% and specificity of 79% [19]. PubMed was searched from January 1966 to 26 July 2010 and EMBASE from January 1980 to 26 July 2010. A combination of the phrases 'streptococcal pharyngitis' and 'sore throat' (maps to: (('streptococcus'[MeSH Terms] OR 'streptococcus'[All Fields] OR 'streptococcal'[All Fields]) AND ('pharyngitis'[MeSH Terms] OR 'pharyngitis'[All Fields])) OR ('pharyngitis'[MeSH Terms] OR 'pharyngitis'[All Fields] OR ('sore'[All Fields] AND 'throat'[All Fields]) OR 'sore throat'[All Fields])' were entered into the filter. The search was limited by using a combination of phrases for ambulatory care; 'general practice', 'family practice', 'emergency department' and 'primary care' (maps to: ('general practice'[MeSH Terms] OR ('general'[All Fields] AND 'practice'[All Fields]) OR 'general practice'[All Fields]) OR ('family practice'[MeSH Terms] OR ('family'[All Fields] AND 'practice'[All Fields]) OR 'family practice'[All Fields]) OR (('emergencies'[MeSH Terms] OR 'emergencies'[All Fields] OR 'emergency'[All Fields]) AND department[All Fields]) OR ('primary health care'[MeSH Terms] OR ('primary'[All Fields] AND 'health'[All Fields] AND 'care'[All Fields]) OR 'primary health care'[All Fields] OR ('primary'[All Fields] AND 'care'[All Fields]) OR 'primary care'[All Fields])). The search was supplemented by hand checking references of filtered papers, searching Google Scholar, the Cochrane Library and the MEDION database (University of Maastricht). No restrictions were placed on language.

### Study selection

Two investigators (JA and KOB) independently evaluated the title, abstract and subsequently full text of all articles for inclusion and any disagreements were resolved by discussion with a third investigator (WSC). Studies were included if participants were recruited upon first presentation from an ambulatory care setting, had a sore throat as their main presenting complaint, and were ≥ 15 years of age. Both prospective and retrospective studies were included in the review.

Each included study assessed the diagnostic accuracy of signs and symptoms and/or validated the Centor score. The reference standard for all studies was a throat culture. If this information was not available in publications, data were sought from corresponding authors. The majority of studies separated positive results for group A β-haemolytic streptococcal infection from non-group A infection (mostly group C and G). Patients who were positive with a non-group A streptococcal infection were counted as negatives when the data were pooled. Additional file 1 has more information on the reported proportions of non-group A infection.

### Data extraction and quality assessment

Data were extracted by two investigators (JA and KOB) independently, and any discrepancies were resolved by discussion.

**Figure 1:**
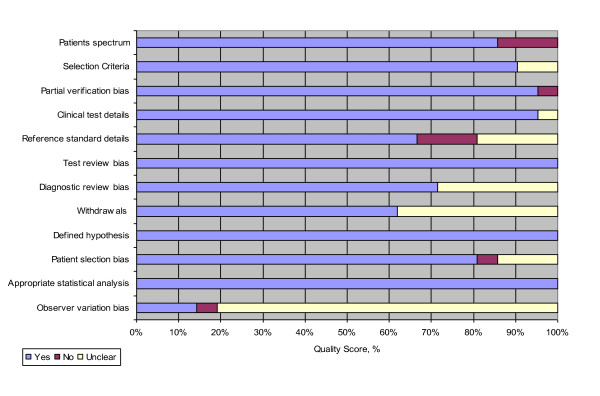
**Reviewer judgements of methodological quality of included studies according to use of modified Quality Assessment of Diagnostic Accuracy Studies (QUADAS) tool**.

The Quality Assessment of Diagnostic Accuracy Studies (QUADAS) tool was used to assess the quality of each included study [21]. This tool was modified to ensure appropriateness to this study. Items 3, 4, 6, 7, 12 and 13 were omitted from the original QUADAS tool as they were not relevant to this study and four questions extracted from other reviews were added; 'Was the hypothesis clearly defined', 'Were the patients selected in a non-biased manner', 'Were the statistical tests for the main outcomes adequate' and 'Were data on observer variation reported and within acceptable range' (Figure 1) [22,23]. Quality assessment was performed independently by three researchers (JA, KOB and WSC). Each article was assessed by at least two researchers with disagreements resolved by review and discussion with the third researcher.

### Data synthesis and analysis

#### Diagnostic accuracy of signs and symptoms

Data were extracted and 2 × 2 tables constructed for the following signs and symptoms: (i) absence of cough, (ii) fever, (iii) anterior cervical adenopathy, (iv) tender anterior cervical adenopathy, and (v) any exudates (either tonsillar exudate or pharyngeal exudate or any exudate). Although some studies examined other signs and symptoms, those chosen for inclusion in this diagnostic test accuracy study were the most consistently studied signs and symptoms.

Review Manager v.5.0.16 [24] and a bivariate random effects model [25] were used to analyse the extracted data. The analysis consisted of (a) summary sensitivities and specificities calculated for each sign and symptom, (b) positive and negative likelihood ratios and (c) summary receiver operating characteristic (SROC) curves. The bivariate random effects model accounts for the bivariate nature of sensitivity and specificity as well as the within-study and between-study variability [25]; as this approach is not available in Review Manager v.5.0.16, the Stata package metandi [26] was used for this part of the analysis.

#### Diagnostic accuracy of the Centor score

As the Centor score is recommended by guidelines as a decision aid for empirical antibiotic use [4,17], we explored the diagnostic accuracy of the score at different cut points. In all, 12 studies were included in this analysis [14,27-37]; 3 studies were excluded from this analysis as they excluded patients with a Centor score less than 2 [38-40]. The analysis consisted of (a) summary sensitivities and specificities and (b) positive and negative likelihood ratios, calculated using a random effect bivariate model (using the Stata package metandi [26]). Post-test probabilities are presented for the Centor score at a range of pretest probabilities.

#### Calibration of the Centor score

We assessed calibration of the Centor score across four levels (0-1, 2, 3 and 4). Calibration enables visual and quantitative assessment of how well a CPR performs across different levels of risk [41]. The predicted number of patients with GABHS pharyngitis (based on the probability calculated in the derivation study [16], Table 1) were compared with the observed number of patients with GABHS pharyngitis in each validation study. The data were pooled and analysed using a Mantel-Haenszel random effects model and risk ratios (RRs) reported. The total heterogeneity across studies was quantified using the I^2 ^index. The Centor score data were analysed in groups (score 0-1, 2-3 and 4) as the ACP/ASIM guidelines recommend treatment options on the basis of these categories [17]. In the majority of studies, data were available for all score categories; the predicted was calculated for each score category and the results added together to form the group data (0-1, 2-3). For example, Atlas *et al. *[27] reported 11 patients had a score of 0 and 44 had a score of 1. We calculated the number predicted to have GABHS pharyngitis based on the probabilities reported in Table 1, 11 × 2.5% + 44 × 6.5% = 0.275 + 2.86 = 3.135. In one case [29], data were only available for the score group (0-1, 2-3); in this case the mean post-test probability for the group (mean of 2.5% and 6.5% = 4.5%) was used to calculate the predicted score.

We carried out a subgroup analysis to discover the influence of disease prevalence on the performance of the score (cut point 17.1% prevalence as in the derivation study [16]). Poses and colleagues suggested the use of the likelihood ratio formulation of Bayes' theorem to adjust for prevalence [42]. In our review, the method of Poses *et al. *was applied to the meta-analysis data and the effect on the results is discussed.

The Preferred Reporting Items for Systematic Reviews and Meta-analyses (PRISMA) statement was followed during the course of this study [43].

## Results

### Study identification

**Figure 2:**
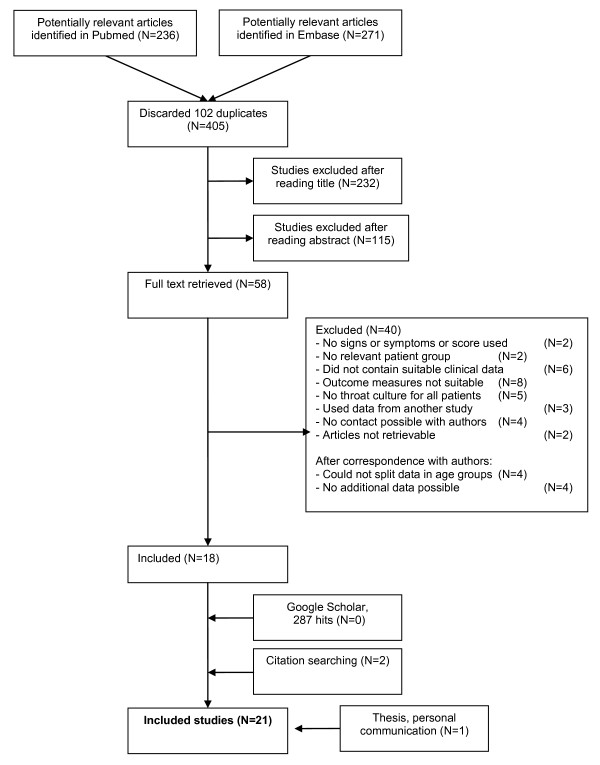
**Flow diagram of studies included in the review**.

A flow diagram of our search strategy is presented in Figure 2. Two researchers screened all potential articles. They agreed that the full text of 58 articles should be examined. In all, 35 relevant studies were identified, 18 of which included only adults whilst the other 17 included both adults and children. Only 4 of the 18 adult only studies reported all required data [16,28,39,44]. The authors of the remaining adult papers were written to for additional data. In all, 13 authors responded and 8 studies were subsequently included [13,27,29,37,38,40,45,46]. After writing to the authors of mixed adult and children papers, 13 responses were received and the data for adults only were included for 8 of these studies [14,31-36,47]. Data from one thesis was included in the analysis and was obtained through a personal communication [30].

Data on signs and symptoms were available in all of the 21 included studies, which included 4,839 patients [13,14,16,27-40,44-47]. A total of 15 studies also provided data on the Centor score, which included 2,900 patients [14,27-40]. The characteristics of each included study are summarised in Additional file 1.

### Study description

The included studies came from a variety of settings and countries. In all, 19 of the studies took place in a primary care setting; the other 2 studies took place in an emergency department setting [16,44]. Five studies were based in the USA [13,16,27,44,46], while nine were based in Europe [28,31,33,35,38-40,45,47], three in Canada [14,34,36], two in New Zealand [30,32], and one each in Thailand [37] and Israel [29].

The prevalence of GABHS pharyngitis in the studies was highly variable, ranging from 4.7% [40] to 37.6% [38]. Three studies excluded all patients with a Centor score less than 2 [38-40]. Three studies included patients with broader presenting symptoms of upper respiratory tract infection [14,37,47].

### Study quality

The result of the quality assessment is shown in Figure 1. The overall quality of the included studies was good. The spectrum of patients was generally appropriate and representative of the patients who would receive the test in practice, the selection criteria were stated and the signs and symptoms being studied were generally clearly described. Quality items on test and diagnostic review bias scored well. This was due to the result of the throat culture being unknown at the time of the first visit when the signs and symptoms were recorded, and the throat culture being analysed by an independent laboratory. Observer variation in assessing signs and symptoms (question 12) was poorly reported.

### Diagnostic accuracy of individual symptoms and signs

The sensitivity, specificity, positive likelihood ratio (+ LR) and -LR are reported in Table 2. Absence of cough and tender cervical adenopathy had a higher sensitivity than specificity (sensitivity 0.74, specificity 0.49 and sensitivity 0.67, specificity 0.59 respectively), while fever and any exudates had a higher specificity than sensitivity (sensitivity 0.50, specificity 0.70 and sensitivity 0.57 and specificity 0.74 respectively). 'Any exudates' had the highest positive likelihood ratio (LR) (2.20), suggesting it raises the probability of disease by 15% to 20% when present [48]. Absence of cough and tender anterior cervical adenopathy both decrease the likelihood of GABHS pharyngitis by 15% to 20% when absent.

**Table 2:** Summary estimates of sensitivity, specificity, positive likelihood ratio (LR) and negative LR calculated for signs and symptoms using a bivariate random effects model

Sign or symptom	No. of studies	No. of patients	Sensitivity (95% CI)	Specificity (95% CI)	+LR (95% CI)	-LR (95% CI)
Absence of cough	19	4,653	0.74 (0.68 to 0.79)	0.49 (0.40 to 0.58)	1.46 (1.28 to 1.66)	0.53 (0.46 to 0.61)

Fever^a^	21	4,635	0.50 (0.39 to 0.62)	0.70 (0.58 to 0.79)	1.65 (1.40 to 1.95)	0.71 (0.64 to 0.80)

Anterior cervical adenopathy^b^	9	2,101	0.65 (0.55 to 0.74)	0.55 (0.45 to 0.64)	1.45 (1.25 to 1.67)	0.63 (0.52 to 0.76)

Tender anterior cervical adenopathy^b^	16	4,144	0.67 (0.52 to 0.79)	0.59 (0.49 to 0.69)	1.65 (1.41 to 1.92)	0.56 (0.41 to 0.76)

Any exudates^c^	21	4,839	0.57 (0.44 to 0.70)	0.74 (0.63 to 0.82)	2.20 (1.76 to 2.74)	0.58 (0.47 to 0.72)

^a^The most widely used cut-off point to indicate fever was 38.0°C. Studies also used 37.5°C [40], 37.8°C [37,44,46], 38.3°C [13], 38.5°C [31,45].

^b^Studies reported on tender lymph nodes, adenopathy or tender adenopathy. All adenopathy results were categorised into tender anterior cervical adenopathy or anterior cervical adenopathy.

^c^Includes results for 'tonsillar exudate', 'pharyngeal exudate' and 'exudate'. If both tonsillar and pharyngeal exudate were reported only the results for tonsillar exudate were added to avoid double counting.

**Figure 3:**
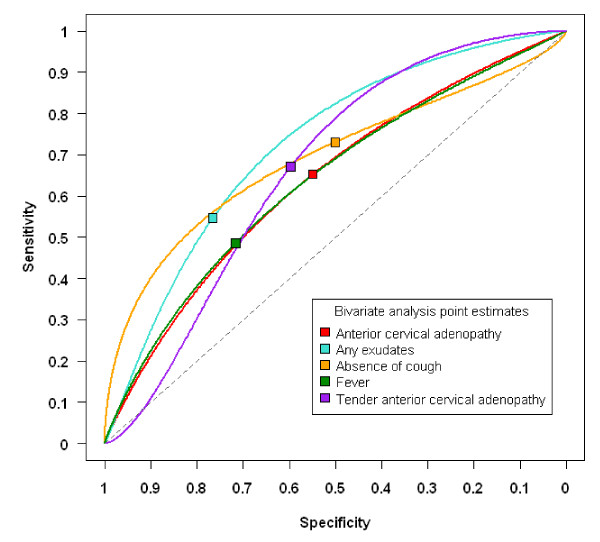
**Summary receiver operating characteristic (ROC) curve for signs and symptoms**.

A summary ROC curve for signs and symptoms is presented in Figure 3. The curve and point estimate are presented as a strong negative correlation was found between sensitivity and specificity for some of the signs and symptoms, suggesting the presence of an implicit threshold effect. The ROC curves are overlapping, suggesting that each of the individual signs and symptoms included in the analysis have a similar, relatively low ability to discriminate GABHS pharyngitis patients from other patients presenting with a sore throat.

### Diagnostic test accuracy of the Centor score

Summary estimates for the four levels of Centor categories show (as expected) increasing specificity and diminishing sensitivity with higher scores (Table 3). When ≥ 3 signs or symptoms are present (the recommended cut-off point for empirical antibiotic treatment according to the ACP/ASIM guidelines), the Centor score has a specificity of 0.82 and a sensitivity of 0.49 and raises the probability of GABHS in absolute terms by 17% in situations of intermediate pretest probability (pretest probability 15%) (Table 4) [48]. Based on the pooled results, Table 4 shows the post-test probability of GABHS pharyngitis for a range of pretest probabilities. If clinicians estimate the prevalence of GABHS pharyngitis in their area, this table can be used to find the corresponding post-test probability of GABHS.

**Table 3:** Summary estimates of sensitivity, specificity, positive likelihood ratio (LR) and negative LR for the Centor score, calculated using a bivariate random effects model

Centor score	No. of studies	Sensitivity (95% CI)	Specificity (95% CI)	+ LR (95% CI)	- LR (95% CI)
≥ 1	11	0.95 (0.91 to 0.97)	0.18 (0.12 to 0.26)	1.16 (1.08 to 1.25)	0.27 (0.16 to 0.46)

≥ 2	12	0.79 (0.71 to 0.86)	0.55 (0.45 to 0.65)	1.76 (1.51 to 2.07)	0.37 (0.29 to 0.48)

≥ 3	11	0.49 (0.38 to 0.60)	0.82 (0.72 to 0.88)	2.68 (1.92 to 3.75)	0.62 (0.52 to 0.74)

4	11	0.18 (0.12 to 0.27)	0.95 (0.92 to 0.97)	3.85 (2.05 to 7.24)	0.86 (0.78 to 0.93)

**Table 4:** Post-test probability of group A β-haemolytic streptococcal (GABHS) pharyngitis

Points	Likelihood ratio	Pretest probability of GABHS pharyngitis (%)							
		
		5	10	15	20	25	30	35	40
≥ 1	1.16	6	11	17	22	28	33	38	44

≥ 2	1.76	8	16	24	31	37	43	49	54

≥ 3	2.68	12	23	32	40	47	53	59	64

4	3.85	17	30	40	49	56	62	67	72

### Calibration of the Centor score

**Figure 4:**
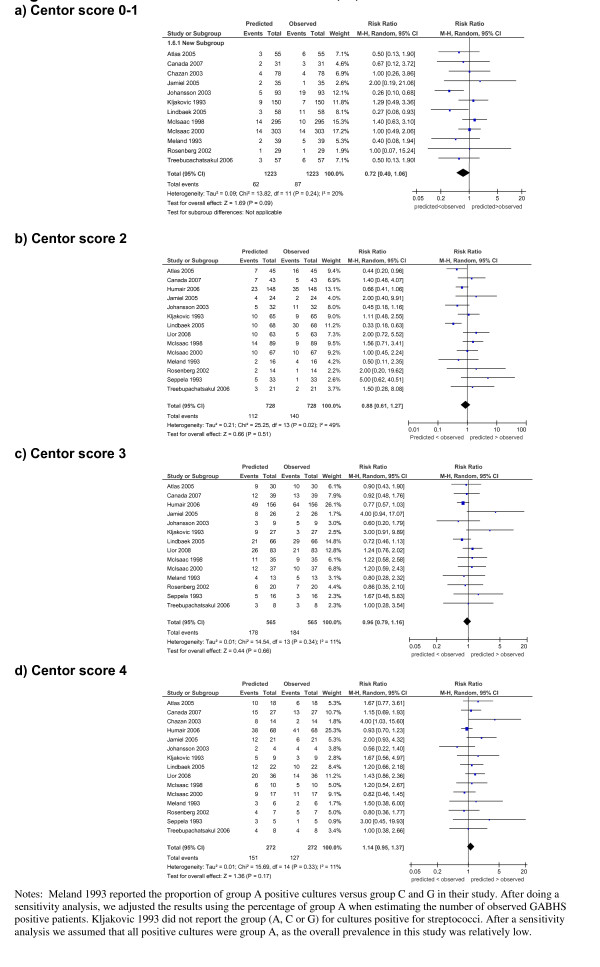
**Forest plots of Centor scores 0-1, 2, 3 and 4**.

There was no significant difference between predicted and observed events in any of the Centor score categories (Figure 4), suggesting that the Centor score performed as well in the pooled data at predicting the probability of culture positive GABHS pharyngitis across the strata of risk as it did in the derivation study. Slightly fewer events were predicted in the 0-1 category than observed (z = 1.69, *P *= 0.09). There was modest between-study heterogeneity in the analysis, with I^2 ^values ranging from 11 to 49%.

A subgroup analysis based on prevalence was carried out for each score category of the Centor score. The prevalence was classified as 'high' if it was higher than that reported in the Centor derivation study (17.1%). The analysis showed that in the 0-1 and 2-3 score categories fewer events were predicted than observed in the high prevalence subgroup (0-1 n = 7 RR 0.42, 95% CI 0.25 to 0.70; 2-3 n = 9 RR 0.77, 95% CI 0.60 to 0.98) and slightly more events were predicted than observed in the low prevalence subgroup (0-1 n = 5 RR 1.11, 95% CI 0.72 to 1.71; 2-3 n = 6 RR 1.43, 95% CI 1.07 to 1.91). For score category 4, prevalence made little difference to the performance of the score (high prevalence n = 9 RR 1.13, 95% CI 0.89 to 1.43 and low prevalence n = 6 RR 1.20, 95% CI 0.85 to 1.70). Overall the subgroup analysis reduced interstudy heterogeneity, but did not improve the performance of the score.

We used the method of Poses *et al. *[42] to adjust each study for its own prevalence. We found this method decreased between-study heterogeneity, but the predicted-to-observed ratio did not improve significantly (data not shown).

## Discussion

### Principal findings

From the diagnostic test accuracy of signs and symptoms analysis, all symptoms and signs included in the analysis have only a modest ability to discriminate patients with GABHS pharyngitis from those without it (range +LR 1.45-2.20, range -LR 0.53-0.71); therefore no sign or symptom on its own has the power to rule in or rule out a diagnosis of GABHS pharyngitis. Fever and 'any exudates' have a higher specificity than sensitivity and are more valid for ruling in a diagnosis of GABHS pharyngitis when present, while absence of cough and tender anterior cervical adenopathy have a higher sensitivity than specificity and are more valid for ruling out GABHS pharyngitis when absent. Based on our analysis it could be argued that the signs and symptoms present in the Centor score could be given different weights depending on whether the aim of the physician is to rule in or rule out a diagnosis of GABHS pharyngitis. However, it is highly unlikely that the benefit would outweigh the cost of complicating such a simple score.

In terms of diagnostic accuracy, our analysis of the Centor score as a decision aid for antibiotic prescribing suggests that although the score is reasonably specific when ≥ 3 signs or symptoms are present (0.82) and very specific when 4 are present (0.95), the post-test probability of GABHS pharyngitis is relatively low (that is, for a prevalence of 15% and a score of ≥ 3, post-test probability is 32%, Table 4). Therefore, although the Centor score can enhance appropriate prescribing of antibiotics, it should be used with caution as treating all patients presenting with a sore throat and a score of ≥ 3 may lead to many patients being treated with antibiotics inappropriately (Table 4).

In terms of calibration, the Centor score produces consistent observed:predicted performance across all risk strata in different populations (Figure 4). This shows that the Centor score is well calibrated, suggesting that the rule is generalisable across settings and countries [41].

### Findings in the context of other studies

The diagnostic accuracy of signs and symptoms findings of this systematic review are consistent with a previous review on GABHS pharyngitis which concluded that no sign or symptom on its own is powerful enough to rule in or rule out the diagnosis of GABHS pharyngitis [12]. Not all studies reported the same signs or symptoms to be of similar predictive value. For example, Lindbaek *et al. *and Llor *et al. *found that among the four Centor criteria, only cervical adenitis and absence of cough were significantly more frequent in the GABHS pharyngitis patients compared to those with negative cultures [33,39], while Meland *et al. *found that tonsillar exudate had no predictive ability [35]. Our meta-analysis shows that all individual symptoms and signs that comprise the Centor score do have modest discriminatory power, with 'any exudates' being the strongest (Table 2).

To the best of our knowledge, this is the first diagnostic test accuracy review of the Centor score. Wigton *et al. *[49] reported that a cut-off point of ≥ 2 signs or symptoms in their patient cohort produced a sensitivity of 86% and a specificity of 42%, which was similar to our pooled results (79% and 55% respectively). The most appropriate cut point for antibiotic treatment when using the Centor score depends on the clinicians aim; adults in Western society rarely have complications such as rheumatic fever and clinicians may want to ensure a high specificity in the test, which would lead to lower antibiotic prescription rates but missed cases of GABHS pharyngitis. Where as a clinician in a developing country with a high rate of rheumatic fever, and no access to other diagnostic tests, may feel a high sensitivity is more important.

### Strengths and weaknesses

The strengths of this study include the inclusion of additional data from authors, and pooling the results of validation studies for the Centor score so that formal quantitative validation of the Centor score is accomplished.

We acknowledge that our review has several limitations: there is moderate heterogeneity in the Centor score calibration analysis (I^2 ^= 11% to 49%). Heterogeneity in the studies could be due to a variety of factors: chance; a threshold effect as caused by observer variation in the measurement of signs and symptoms; a variation in the pretest probability of GABHS pharyngitis; or other unanticipated factors. The prevalence of GABHS pharyngitis was highly variable between studies (Additional file 1). We addressed the effect of study prevalence as a source of heterogeneity in our calibration analysis.

Although we used a systematic search strategy, we acknowledge that it was not exhaustive and it is possible that we may have missed relevant articles. In particular, the use of search filters in systematic reviews is debatable and not always recommended [50].

The use of a throat culture as the reference standard for diagnosing GABHS pharyngitis is open to some debate. To date, throat culture is still considered by most to be the reference standard of choice when diagnosing GABHS pharyngitis [3,8]. Newly developed RADTs can be used in ambulatory care settings, with results available within minutes [51,52]. However, throat cultures and RADTs fail to distinguish between active infection and carriage, which can lead to inappropriate prescribing of antibiotics for cases of carriage [10,53]. In addition, many argue that lower sensitivities and the lack of cost effectiveness of RADTs in primary care, will limit their use and that signs and symptoms will always be valuable [54,55].

The method of analysis in pooling the individual Centor score studies (calibration analysis) is based on the comparative approach used by Bont *et al. *to validate the CRB-65 CPR in a single validation study [56]. This method extends and employs the absolute risk from the derivation study as a model to generate predicted values in subsequent validation studies. The absolute risk is presented in CPR risk strata so that the clinical value of the CPR across these strata can be assessed. Our method is further supported by an explorative analysis (unpublished results) that compares our original method to a validated and published method for comparing predicted-to-observed values [57]. No statistically significant difference was found between the predicted events by the two methods (*P *> 0.05). A limitation of this method is that it compares the proportion of patients predicted and observed to have GABHS pharyngitis but without patient level data it is not possible to determine if the positives as predicted by the Centor score are the same patients who are positive based on the throat swab.

### Implications for practice

Our meta-analysis of Centor score suggests that it transfers well to other populations and can be used by clinicians to make informed decisions (Table 4 and Figure 4). However, the relatively low post-test probability of GABHS pharyngitis even in areas of high prevalence (Table 4), suggests the score should be used with caution by clinicians when used as a decision aid for antibiotic prescribing. Studies have shown that the use of scores can improve antibiotic prescribing [14], while others have found them no better than clinician judgement [58].

A barrier when introducing CPRs such as the Centor score into practice is that clinicians often fail to apply them [59,60]. One community-based study that used repeated clinical prompts for the modified Centor score to try and influence physician's behaviour when prescribing antibiotics for sore throats, found no significant change in physician behaviour [60]. However, the authors had problems retaining community-based physicians for the duration of the study and believe their results may have been biased by these losses [60].

The formal incorporation of CPRs can be facilitated by computer-based clinical decision support systems (CDSSs) that quantify diagnostic and prognostic information so as to provide physicians with patient specific recommendations: such aids have been shown to reduce antibiotic prescribing in respiratory tract infections in children in primary care settings [61,62].

## Conclusions

Individual symptoms and signs have only a modest ability to rule in or out a diagnosis of GABHS pharyngitis. The Centor score uses a combination of signs and symptoms to predict the risk of GABHS pharyngitis; the score is well calibrated across a variety of countries and settings. It has reasonably good specificity, and can enhance the appropriate prescribing of antibiotics, but should be used with caution in low prevalence settings of GABHS pharyngitis such as primary care.

## Competing interests

The authors declare that they have no competing interests.

## Authors' contributions

All authors were responsible for initiating the research and writing the study protocol. JA, KKO'B, CT and BDD performed the statistical analysis. JA and KKO'B wrote the first draft of the paper and all authors were involved with commenting on subsequent drafts of the paper. TF is the guarantor.

## Pre-publication history

The pre-publication history for this paper can be accessed here:

http://www.biomedcentral.com/1741-7015/9/67/prepub

## Supplementary Material

Additional file 1 **Table S1**. Summary of included studies. Click here for file
