# Supplementary material for: Predicting streptococcal pharyngitis in adults in primary care: a systematic review of the diagnostic accuracy of symptoms and signs and validation of the Centor score
Source: BMC Med. 2011 Jun 1;9:67. doi: 10.1186/1741-7015-9-67 (PMC3127779; doi:10.1186/1741-7015-9-67)
Supplement: Additional file 1 — Table S1. Summary of included studies. [file 1741-7015-9-67-S1.DOC]

Additional Files:

Table S1

| ***Study*** | ***Prior*** | ***Setting*** | ***Notes*** |
| --- | --- | --- | --- |
| Atlas et al.  2005^27^  N=148 | 25.7% | Prospective study with adults ≥ 18 yrs and presenting with symptoms of acute pharyngitis in a primary care settings at Massachusetts General Hospital between July 2002 and June 2003.  Population: 16.7% male, mean age 35.6 years (SD 10.9) |  |
| Canada et al.  2007^28^  N=140 | 24.3% | Patients ≥ 14 yrs presenting with the main symptom of a sore throat to an urban primary care health center in Madrid, Spain between Feb and May 2005.  Population: 37.9% males, mean age 32.1 years (SD 14.8), range 14 – 81 years | Paper in Spanish |
| Centor et al.  1981^16^  N=234 | 17.1% | Adults > 15 yrs presenting to Medical College of Virginia ER with a sore throat from Feb 15 to April 15 1980. | Derivation of the Centor score |
| Chazan et al.  2003^29^  N= 207 | 24.2% | Prospective study of adults > 16 yrs presenting with a sore throat at primary care clinics of the Clalit Health Services in Nazareth, Israel, from Dec 1999 to March 2000 | -207 patients included in diagnostic test accuracy of signs and symptoms analysis.  -204 patients in analysis of Centor score analysis. |
| Gulich et al 2002^44^  N=381 (two study phases, phase 1 n=116, phase 2 n=265) | 27.9% (Phase 1 28.7%, Phase 2 27.5%) | Both phases of the study were prospective and involved adults >15 yrs presenting with a newly developed sore throat at a family medical practitioners office in Germany.  Phase 1 population: 57.0% male, median age 33 years, range 16 - 68 years  Phase 2 population: 50.2% male, median age 32 years, range 16 – 76 years |  |
| Heckerling et al. 1985^43^  N=111 | 19.8%  *** | Retrospective study of adults ≥ 18 yrs who were discharged from emergency services with a diagnosis of pharyngitis, between April 1 and June 30, 1982. Chicago, USA | This study presented results on the basis of positivity for β hemolytic streptococci regardless of serotype. The authors reported that 73% of the positive cultures were group A and we adjusted their results based on this |
| Humair et al.  2006^38^  N=372 | 37.6% | Patients ≥ 15 yrs with pharyngitis with a Centor score ≥2, presenting to a walk-in clinic of a university-based primary care center in Geneva, Switzerland from Mar 1 1999 to Sep 2001.  Population: 44.9% male, mean age 29.6 years (SD 9.5) |  |
| Jamiel et al 2004^30^  N = 107** | 10.3% | Patients presenting with a sore throat to a family medical practitioner in New Zealand.  For adult data only: 63.6% male, mean age 33 years (SD 13), range 15 – 65 years  For group A positive group: 11 people, 64% male, mean age 24 years (SD 5.8)  For non group A positive group: 6 people, 33% male, mean age 27.8 (SD 6.0)  For culture negative group: 90 people, 65% males, mean age 34.5 years (SD 13.7) | Included children in original study (48% <15 years). |
| Johansson et al. 2003^31^  N=138** | 28.3% | Consecutive patients presenting with sore throat from 3 health care centers in Malmo, Sweden in 2 winter months in 2001.  For whole group: 44% male | This study included 138 adults out of the 169 patients who participated in the study. |
| Kljakovic et al. 1993^32^  N= 251** | 8.8% | Prospective study of consecutive patients presenting with sore throat as primary complaint in a suburban family medical practice in Karori Valley, New Zealand, from mid November 1988 till June 1990.  For adult data only: 46.6% male, mean age 31.7 years (SD 14.6), range 15 – 88 years.  For group with positive cultures: 22 people, 31.8% male, mean age 26.2 years (SD 9.89), range 15 – 56 years.  For group with negative cultures: 229 people, 44.1% male, mean age 32.2 years (SD 14.9), range 15 – 88 years | This study included both children and adults, there were 251 adults.  The authors did not differentiate group A from non group A streptococci positive patients |
| Komaroff et al. 1986^45^  N=693 | 9.7% | Walk-in adult (≥16yrs) patients complaining of a sore throat at 2 health maintenance organisations & 2 hospital-based ambulatory care practices, from winter 1977 to winter 1978. Boston and Rhode Island, USA.  Population: 38% male, mean age 30.9 years (SD 10.5) |  |
| Lindbaek et al. 2005^33^  N=217** | 36.8% | Prospective study of patients who presented with a sore throat in two health centers, in Stokke and Kongsberg, Norway, from April 2000 till June 2002.  -For whole group: 40% male, mean age 23.9 years (95% CI 22.2 – 25.6).  -Group A positive group: 127 people, 38% male, mean age 21.5 years (95% CI 19.0 – 23.9)  -Group C or G positive group: 33 people, 39% male, mean age 25.2 years (95% CI 20.6 - 29.7)  -Culture negative group: 146, 43% males, mean age 5.8 years (95 CI 23.2 – 28.4) | This study included 244 adults out of 306 patients enrolled in the study.  The authors included details of the proportion of group A streptococci positive patients compared with group C and G. |
| Llor et al 2008^39^  N = 182 | 22.0% | Patients over the age of 14 years presenting with a sore throat to a family practice clinic in Spain.  Population: 36.3% male, average age 30.6 (SD12.1 years)  Only included patients with 2 or more Centor criteria | Paper in Spanish  -22.0% had group A streptococcal  -4.5% had group B  -14.3% had group C  -3.4% had group F  -1.2% had group G |
| McIsaac et al.  1998^14^  N = 423** | 8.9% | Patients aged 3 years or more who presented with new URTI in a Family Medicine Center at Mount Sinai Hospital in Toronto, Canada, from December 1995 to February 1997  For whole population: 29.4% male  For group with positive cultures: 41.7% male  For group with negative cultures: 17.2% male | This study included 423 adults out of 521 patients enrolled in the study.  The study derives and validates the McIsaac score (or modified Centor score) |
| McIsaac et al.  2000^34^  N = 453** | 10.7% | Patients aged 3 yrs or more who presented with a new URTI and sore throat to 97 family physicians in 49 Ontario communities, Canada, in fall 1998  For whole group: 32.7% male | This study included 453 adults out of 620 patients enrolled in the study  This study validates the McIsaac score (or modified Centor score) |
| Meland et al.  1993^35^  N=77** | 20.8%  *** | Consecutive patients who presented with tonsillopharyngitis in a health center in Bergen, Norway, in 1988/89. | This study included 77 adults out of 133 patients enrolled.  The authors did not separate group A from group C and G streptococci in their analysis.  For whole group:  Group A prevalence = 18.0%  Group C & G = 10.5% |
| Rosenberg et al. 2002^36^  N=70** | 20.0% | Patients presenting with a sore throat in two community emergency departments in Toronto, Canada, between Jan 1999 and Feb 2000. | This study included 77 adults out of 126 patients enrolled. |
| Seppala et al.  1993^40^  N=106 | 4.7% | Adult patients with a main complaint of sore throat presenting to a private family medical practice health center in Finland from Jan to Mar 1986. | Prevalence of group A = 4.7%  Prevalence of group C = 12.3%  Prevalence of group G = 4.7% |
| Solak et al.  2005^46^  N=95** | 6.3% | Patients presenting with URTI complaints in outpatient clinics in Ankara, Turkey. | This study included 95 adults out of 276 patients enrolled.  Article in Turkish  Patients presented with URTI not specifically sore throats |
| Treebupachatsakul et al.  2006^37^  N= 94** | 16.0% | Prospective study adult outpatients presenting with an URTI to a Bangkok Hospital, Thailand from Apr to Oct 2004.  For whole group: 30.1% male, mean age 32.9 years | This study included only the 94 adult patients with pharyngitis out of the 292 patients enrolled. |
| Walsh et al.  1975^13^  N=418 | 15.3% | Adult (>15 years) patients seen between Aug 1973 and May 1974, in an ambulatory clinic. All patients presented with URT complaints. New Hampshire, USA.  Population: 34.7% male | There are 3 patients under the age of 15 included in this study; all 3 patients had negative cultures. |

** This is the number of patients from the study that were included in our analysis. Exclusions were based on our inclusion criteria

and the majority were due to age <15 years

*** This is the actual prevalence of group A β-hemolytic streptococcal pharyngitis, after exclusion of group C and G
